# Prostate cancer among Saudis: a registry review

**DOI:** 10.1097/MS9.0000000000001448

**Published:** 2023-11-07

**Authors:** Ahmed Alasker, Tarek Z. Arabi, Mohammad A. Alghafees, Belal N. Sabbah, Saleha Abdul Rab, Abdulrahman K. Alageel, Ahmed Emad Abouelkhair, Abdulmoiz Kaiser Abdulwali, M. Mohanad Imad Al Hennawi, Waleed Fallatah, Ziyad F. Musalli, Yasser A. Noureldin

**Affiliations:** a College of Medicine, King Saud Bin Abdulaziz University for Health Sciences; b Division of Urology, King Abdulaziz Medical City; c College of Medicine, Alfaisal University; d King Abdullah International Medical Research Center; e College of Medicine, King Saud University, Riyadh, Saudi Arabia

**Keywords:** cancer incidence, distant metastasis, factors associated with mortality, prostate cancer, Saudi Arabia

## Abstract

**Background:**

Policy makers in Saudi Arabia greatly rely on published studies to make major public health decisions. Prostate cancer (PCa) studies in Saudi Arabia are either outdated or limited to local regions.

**Aim:**

The authors aim to analyze the Saudi Cancer Registry to determine the incidence of PCa across all regions of the Kingdom and the risk factors of poor prognosis in the population.

**Methods:**

Patients diagnosed with primary PCa from 1 January 2008 to 31 December 2017 were included in the study from the Saudi Cancer Registry. Incidence rates and risk factors for poor survival were calculated.

**Results:**

A total of 3607 PCa patients were retrieved. PCa incidence rates ranged from 0.2 to 1.4 per 100 000. Most of the patients were aged 60 and older (86.5%; *n*=3120), married (97%; *n*=3497) and lived in the central region (38.1%; *n*=1375). The mean age at diagnosis was 71.1 (10.8) years. Over half of all tumors were poorly differentiated (64.2%; *n*=2317), and localized (60.4%; *n*=2180). The all-time metastasis rate reached 31.4% (*n*=1131). The lowest mean survival was in those with distant metastasis (*P*=0.039). Age groups, marital status, tumor morphology, place of residency, and grade were not proven to significantly influence survival.

**Conclusion:**

The high metastasis rate and evidence of a greater incidence of newly diagnosed metastatic PCa indicate that the idea of select screening for certain high-risk populations is not farfetched. The authors encourage the promotion of awareness regarding PCa risk factors and screening to optimize prognosis and minimize late presentations and high metastasis rates.

## Introduction

Highlights

- We analyzed prostate cancer incidence and risk factors in Saudi Arabia from 2008 to 2017, involving 3607 patients.
- The study identified a high metastasis rate (31.4%) and a significant presence of poorly differentiated tumors among older, married men in the central region.
- Our findings suggest selective screening for high-risk populations and increased awareness to minimize late diagnosis and high metastasis rates.

The most frequently identified malignancy and the fourth major cause of cancer-related death in males globally is prostate cancer (PCa)^1^. At early stages, PCa is often asymptomatic and only requires active surveillance^2^. In advanced stages; however, patients may present with obstructive symptoms such as urinary hesitancy and retention^3^. PCa is typically clinically inconsequential for the vast majority of cases, with biopsy-detected PCa prevalence being 30 times higher than PCa-specific mortality^4^.

In Saudi Arabia, the incidence rates of PCa vary largely across the different regions of the country^3^. According to the WHO Globocan Report, 693 new PCa cases were diagnosed and 204 deaths reported across the country in 2020 alone^5^. A recent study by Otifi *et al*.^3^ assessed the prevalence of PCa in the Aseer region of Saudi Arabia. Between 2008 and 2018, the prevalence of PCa in the region was 8.7% among 883 patients. Another retrospective study in Jeddah reported an incidence of 28.5%^6^. Contrarily, a similar study based in Riyadh found that only around 2% of patients had PCa^7^. Overall, these studies indicate that PCa may be a greater concern in certain regions of the Kingdom than others.

Health policy makers in Saudi Arabia greatly rely on published studies to make major public health decisions, such as cancer screening programs^8^. However, current studies in Saudi Arabia are either outdated or limited to local regions. Hence, in this study, we analyzed the Saudi Cancer Registry to determine the incidence of PCa nationwide and across all regions of the Kingdom and risk factors of poor prognosis. This is the most recent analysis of PCa incidence in Saudi Arabia that the authors are aware of.

## Methods

All Saudi patients who were diagnosed with a primary prostate tumor between 1 January 2008 and 31 December 2017, were included in this retrospective chart review. Patients who had been identified as having malignancies that had spread to the prostate were excluded from the investigation. The information was gathered from the Saudi Cancer Registry, which through five regional offices, gathers information from all of Saudi Arabia’s military, private, and civilian hospitals. At the main office in Riyadh, data analysis and periodic reporting were carried out. All of the qualified patients were included. Year of diagnosis, age, marital status, region of the Kingdom, tumor histological subtype, tumor behavior, tumor grade, tumor extent, the basis of the diagnosis, and the prognosis were the factors that were grouped.

With the help of IBM SPSS version 23.0, data analysis was carried out (IBM Corporation). For categorical variables, frequency, and percentage were employed, while a mean and SD were utilized for continuous variables. An ANOVA was used to compare the means of each group, and a *χ* ^2^ was employed to assess connections between the categorical variables. A Tukey post-hoc test was run after the ANOVA to identify the exact group differences. To assess the factors affecting survival and to create survival curves for the various factors, a Kaplan–Meier survival analysis was conducted. The variables predicting patient survival were identified using a Cox proportional hazards regression analysis. The model took into account the following variables as predictors: age, marital status, place of residency, morphology, grade, and extension. The significance level was set at 0.05.

Acinar and nonacinar cell adenocarcinomas incidence rates were determined for each year separately, and the average for all years was then calculated and presented. PCa incidence per 100 000 people on average for each year was also computed. The General Authority for Statistics provided information on the size of the nation’s population for use in calculating the incidence rate for each year. The King Abdullah International Medical Research Center’s Institutional Review Board, which is part of the Ministry of National Guard-Health Affairs in Riyadh, Saudi Arabia, gave its clearance for the study (approval number NRC21R/499/11). The privacy of the patient was protected. Only the research team gathered and used the data. To maintain anonymity, serial numbers were utilized in place of medical record numbers. The necessity for informed consent was eliminated because the study was retrospective in nature and used anonymized patient data.

## Results

In total, 3607 cases of PCa were reported over the designated study period. The majority of the patients were 60 years of age or older, (86.5%; *n*=3120), married (97%; *n*=3497) and lived in the central region (38.1%; *n*=1375) as shown in Table 1. At diagnosis, the mean age was 71.1 (10.8) years.

**Table 1:** Sociodemographic profile of the participants.

Demographical characteristics	*n*=3607 (%)
Age	
18 years and younger	8 (0.20)
19–39 years	6 (0.20)
40–59 years	473 (13.10)
60 years and older	3120 (86.50)
Marital status	
Single	93 (2.60)
Married	3497 (97.00)
Divorced	2 (0.10)
Widowed	15 (0.40)
Place of residency	
Central region	1375 (38.10)
Eastern region	784 (21.70)
Northern region	76 (2.10)
Western region	1064 (29.50)
Southern region	305 (8.50)
International	3 (0.10)
Age	
Mean	71.13
SD	10.80

As shown in Table 2, the predominant subtype was acinar adenocarcinoma (93.5%; *n*=3374). More than half of the tumors were poorly differentiated (64.2%; *n*=2317) and localized (60.4%; *n*=2180). Histology of the primary tumor was the primary diagnostic technique (94.4%; *n*=3405).

**Table 2:** Characteristics of the tumor.

	*n* (%)
Morphology	
Acinar adenocarcinoma	3374 (93.5)
Squamous cell carcinoma	16 (0.4)
Ductal adenocarcinoma	209 (5.8)
Embryonal rhabdomyosarcoma	8 (0.2)
Grade	
Grade I (well differentiated)	142 (3.9)
Grade II (moderate differentiated)	931 (25.8)
Grade III (poor differentiated)	2317 (64.2)
Grade IV (undifferentiated Anaplastic)	217 (6)
Extension	
Localized	2180 (60.4)
Regional: direct extension	232 (6.4)
Regional: lymph node	64 (1.8)
Distant metastasis	1131 (31.4)
Base of diagnosis	
Histology of metastases	115 (3.2)
Histology of primary	3405 (94.4)
Autopsy	87 (2.4)
Year of diagnosis	
2008	41 (1.10)
2009	282 (7.80)
2010	306 (8.50)
2011	327 (9.10)
2012	305 (8.50)
2013	316 (8.80)
2014	348 (9.60)
2015	356 (9.90)
2016	440 (12.20)
2017	411 (11.40)
2018	475 (13.20)

**Figure 1:**
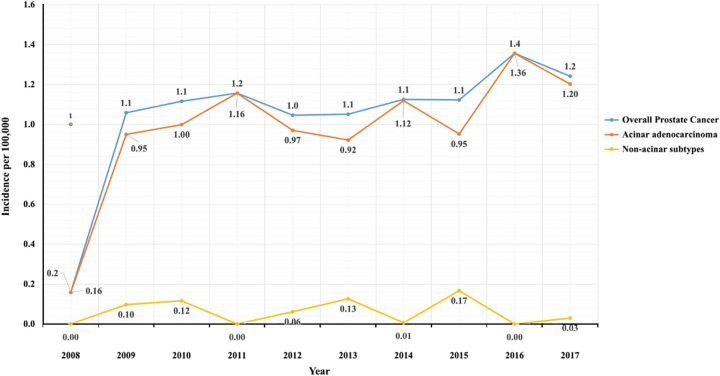
Incidence of prostate cancer per 100 000 throughout the years.

**Figure 2:**
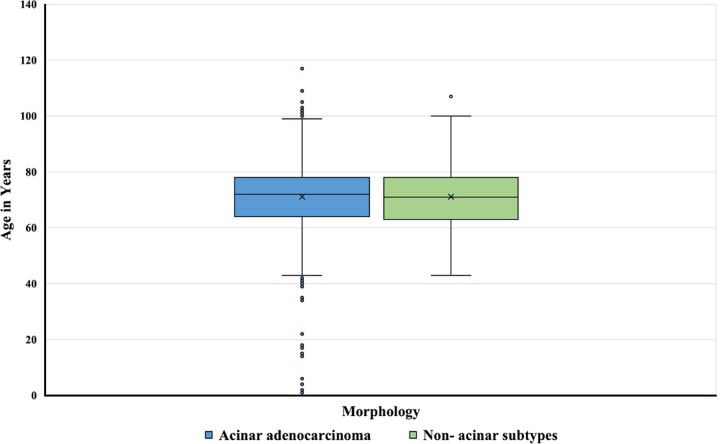
Comparison of age across prostate cancer morphology.

PCa incidence rates varied from 0.2 per 100 000 in 2008 to 1.4 per 100 000 in 2016, as illustrated in Figure 1. Acinar adenocarcinoma followed a similar trend due to it compromising 93.5% of the tumors. The incidence rate of nonacinar subtypes ranged from 0.1 per 100 000 in 2009 to 0.17 per 100 000 in 2015. The overall PCa mortality rate was 14.3%. The mean diagnosis to death interval was 4.45 (2.3) years. In terms of age distribution, there was likewise no difference, as illustrated in Figure 2.

Table 3 demonstrates the Kaplan–Meier survival analysis across different factors. Only extension showed a significant difference in the survival distribution (*P*=0.006). Whereas localized tumors were associated with the highest mean survival, regional lymph node involvement and distant metastases were associated with the lowest mean survival. Age groups, marital status, tumor morphology, place of residency, and grade were not proven to significantly influence survival.

**Table 3:** Kaplan–Meier survival analysis across different factors.

Factor	Mean years of survival	*P*
Age		0.883
40–59 years	4.47	
60 years and older	4.46	
Marital status		0.952
Single	4.8	
Married	4.44	
Place of residency		0.248
Central region	4.45	
Eastern region	4.34	
Northern region	3.33	
Western region	4.65	
Southern region	4.5	
Morphology		0.234
Acinar adenocarcinoma	4.49	
Ductal adenocarcinoma	4.13	
Grade		0.495
Grade I (well differentiated)	4.2	
Grade II (moderately differentiated)	4.46	
Grade III (poorly differentiated)	4.46	
Grade IV (undifferentiated Anaplastic)	4.44	
Extension		0.006*
Localized	4.91	
Regional: direct extension	4.78	
Regional: lymph node	3.98	
Distant metastasis	4	

* Significant at level *P<*0.05.

Table 4 displays the Cox proportional hazards regression analysis (factors influencing diagnosis to death interval). Age, marital status, place of residence, grade of tumor, morphology of tumor, and extension were all included in the model as predictors. The following factor was the only one significantly associated with decreased survival: distant metastasis (*P*=0.039, odds ratio=1.479), where localized PCa was the referent. Age groups, marital status, tumor morphology, place of residency, and grade were not proven to significantly influence survival.

**Table 4:** Cox proportional hazards regression analysis (factors influencing time to death).

Factor	*P*	Odds ratio	CI	
Age	0.851	0.999	0.990	1.009
Place of residency (central region is the referent)				
Eastern region	0.584	0.913	0.659	1.265
Northern region	0.259	1.388	0.786	2.451
Western region	0.519	1.113	0.803	1.542
Southern region	0.200	0.774	0.523	1.146
Marital status (married vs single)	0.998	0.999	0.593	1.683
Morphology (ductal adenocarcinoma vs acinar adenocarcinoma)	0.239	1.250	0.862	1.813
Grade (Grade I (well differentiated) is the referent)				
Grade II (moderately differentiated)	0.340	0.721	0.369	1.411
Grade III (poorly differentiated)	0.210	0.647	0.328	1.278
Grade IV (undifferentiated anaplastic)	0.188	0.631	0.317	1.253
Extension (localized is the referent)				
Regional: direct extension	0.878	0.972	0.68	1.39
Regional: lymph node	0.160	1.29	0.904	1.842
Distant metastasis	0.039*	1.479	1.02	2.145

* Significant at level *P<*0.05.

## Discussion

We used the Saudi Cancer Registry in this work to estimate the prevalence of PCa across Saudi Arabia. According to our results, 3607 patients were diagnosed with PCa from 2008 until the end of 2017, with a yearly incidence rate increasing from 0.2 to 1.4 per 100 000 patients in 2008 and 2016, respectively. The incidence rates in Saudi Arabia remain one of the lowest in the world. For example, Siegel *et al*.^9^ found an age-adjusted incidence rate of 105 per 100 000 patients in 2017 in the United States. In Eastern and Western Asia, the estimated cumulative incidence rates are 13.9 and 26.9 per 100 000 patients, respectively^10^.

In this study, we demonstrate a sevenfold increase in incidence rate from 2008 to 2016. This rise in incidence rate is not in line with incidence trends of other countries. In 44 nations, Culp *et al*. evaluated PCa incidence trends. They discovered that throughout the most recent five data years, rates in the majority of countries stabilized (*n*=35) or decreased (*n*=5)^10^. An increase in incidence rates was only seen in four countries (China, Bulgaria, Belarus, and Slovakia). Although the cause of increased PCa incidence rates in Saudi Arabia and other countries remains unclear, it is hypothesized that increased obesity and unhealthy dietary intake may play a significant role^10^. Additionally, improved documentation and registry reporting may have contributed to the increase in cases witnessed in Saudi Arabia^11^.

The majority of PCa cases were located in the central (38.1%), western (29.5%), and eastern (21.7%) regions of the country, respectively. A previous study by Alghamidi *et al*.^12^ assessing the Saudi Cancer Registry found the peak incidence rates in the eastern, central, and western regions, respectively. Although not statistically significant, our results indicate that patients in the northern region have the lowest mean years of survival in the country. To the authors’ knowledge, there have been no studies assessing PCa metastasis rates in the northern region. Hence, it is crucial for future studies to determine the etiologies behind the decreased survival times in this area.

Our study revealed that 31.4% of PCa patients developed distant metastasis rates. These results are substantially higher than those reported in other developed countries. For example, a recent study utilizing the United States Surveillance, Epidemiology, and End Results Registry found that only 5.6% of PCa patients developed metastasis^13^. This vast disparity in metastasis rates between countries may be caused by a lack of population awareness of PCa symptoms and screening. A 2022 cross-sectional study in the western area of Saudi Arabia identified that 47.5% of participants had poor knowledge regarding PCa risk factors, symptoms, and screening^14^. Generally, cancer screening uptake is low among the Saudi population^15^. For example, 92% of Saudi women greater than 50 years of age report that they have never underwent a mammogram for breast cancer screening^16^. In Jeddah, only 33.4% of women aged 21–65 years have received a pap smear for cervical cancer^17^. Therefore, it is important for policy makers to increase awareness of cancer symptoms and screening policies among the Saudi population.

Upon taking a closer look, it appears that the markedly high metastasis rate is further validated by previously published single-center experiences in the Kingdom. In a study carried out in Saudi Arabia’s eastern area, the metastasis rate among patients with fully validated clinical staging data was 64%^18^. In another study done in the central region of Saudi Arabia, it was identified that out of 52 prostate cases detected, 26.9% of them were metastatic^19^. However, it is worth noting that all the cases compromised of acinar adenocarcinoma, which is known to be less aggressive than its ductal counterpart^20^. This relatively high metastasis rate could be attributed to the fact that primary healthcare centers in Saudi Arabia are generally ill-equipped for PCa screening, diagnosis, and treatment^7^. Therefore, PCa patients are often referred to tertiary care centers, which have long waiting lists and are often located at great distances from their places of residence, requiring dedicated travel times and additional costs. Improving the distribution of the available resources may reduce the high metastasis rate in the country.

Additionally, our results demonstrate distant metastasis to be the only statistically significant factor associated with decreased diagnosis to death interval and greater mortality. To our surprise, other factors such as age group, place of residency, marital status, tumor morphology, and particularly, histological grade, were found to be insignificant predictors of survival. On the one hand, these results contrast significantly with the widely evidenced theory that tumor variables (namely tumor volume, histological grade, and advanced tumor stage) are the most important predictors of PCa mortality^21,22^. Additionally, a meta-analysis of studies in the Asia-Pacific region found that patients greater than 65 years have 37 times the risk of PCa-related mortality compared to patients less than 55 [hazard ratio (HR), 37.3; 95% CI: 24–58.1]^23^. Other risk factors identified in the meta-analysis include a BMI greater than 28 k/m^2^ ^23^. On the other hand, several studies support our finding of distant metastasis being a significant negative prognostic factor for greater mortality in PCa patients^24,25^. Furthermore, we identified that the highest survival was seen in patients with localized tumors, in support of the inverse hypothesis, which is that patients with PCa confined to the prostate have a more favorable prognosis^26^. Unfortunately, no additional data on the predictive factors of mortality among Saudi PCa patients exist currently; greater studies are required to shed light on the factors affecting PCa mortality amongst this population.

For the sake of illustration, we compare our findings of factors associated with mortality to those demonstrated by Frendl *et al*.^26^, who determined the most reliable indicators of 10-year mortality in Americans PCa patients to be the following: greater age at diagnosis, poorer patient-reported health, marital status, and smoking at diagnosis (*P*<0.05)—all of which were shown to individually predict mortality risk. In another study by Zhou *et al*., also conducted on an American population, the most significant factors associated with greater PCa-specific mortality were three: (1) Prostate-specific antigen (PSA) doubling time of less than 3 months following radical prostatectomy (HR, 54.9; 95% CI: 16.7–180), (2) a PSA doubling time of less than 3 months following radiotherapy (HR, 12.8; 95% CI: 7.0–23.1), and (3) a biopsy Gleason score of 8–10, which represents the histological grade (HR, 6.1; 95% CI: 3.4–10.7)^27^. It is unclear why the Saudi population of prostate tumor patients appears to be uninfluenced by all factors other than distant metastasis. This may be because our data was extracted from a national registry, while the aforementioned contrasting results are of cohort studies with select patient groups. Additionally, it is worth noting that nearly one-third to half of all PCa patients will develop comorbidities (i.e. chronic obstructive pulmonary disease, stroke, cardiovascular disease, subsequent development of cancers), which ultimately lead to death^28,29^, confounding proper identification of factors associated with mortality.

Despite the available evidence of a lack of adequate harm-benefit balance to start mass screening^7,30^, the high metastasis rate of PCa, as well as recent evidence suggesting greater incidence of newly diagnosed metastatic PCa^31–33^, indicates that the idea of select screening for certain age groups or populations with established risk factors, such as family history or advanced age, is not farfetched^34,35^.

Our study faces three major limitations, which are registry underreporting and a lack of important parameters, such as history of risk factor exposure or the treatment modality used, which have been reported in several other studies utilizing the registry^35^. Although some studies have hypothesized that Saudi Arabia has low PCa incidence rates due to underreporting, single-center studies (which are more accurate) demonstrated that incidence rates still remain lower than other developed countries^7^. Lastly, the Saudi Cancer Registry fails to collect data regarding patients’ PSA levels and Gleason scores. We recommend that health administrators include these parameters in the Registry to improve the value of the data.

## Conclusion

Overall, this study demonstrates that PCa incidence rates within the Kingdom are increasing; however, they remain much lower than international populations. We strongly encourage public health decision makers to promote public health awareness regarding PCa risk factors, symptoms, and screening throughout the Kingdom of Saudi Arabia to decrease late presentations of the disease and high metastasis rates. Future studies are required to determine the underlying cause behind the increased incidence rates in recent years.

## Ethical approval

Ethical approval was not required due to the public availability of the data.

## Consent

Consent is not required by the Saudi Cancer Registry due to the public nature of the data.

## Sources of funding

This study did not receive funding from any source.

## Author contribution

All authors contributed to the research and/or preparation of the manuscript. All authors read and approved the final manuscript.

## Conflicts of interest disclosure

The authors declare no conflict of interest.

## Research registration unique identifying number (UIN)

researchregistry9536.

## Guarantor

Ahmed Alasker.

## Data availability statement

Data are available upon reasonable request to the corresponding author.

## Provenance and peer review

Not commissioned, externally peer-reviewed.
